# Supplementary material for: Ethical implications of using general-purpose LLMs in clinical settings: a comparative analysis of prompt engineering strategies and their impact on patient safety
Source: BMC Med Inform Decis Mak. 2025 Sep 29;25:342. doi: 10.1186/s12911-025-03182-6 (PMC12481957; doi:10.1186/s12911-025-03182-6)
Supplement: Supplementary file 5 — Supplementary Material 5 [file 12911_2025_3182_MOESM5_ESM.docx]

**Appendix E**

**Data Collection Protocol**

**Statistical Methods and Analysis Procedures for LLM Clinical Reasoning Evaluation**

Emphasis on both statistical significance and clinical significance, with comprehensive effect size reporting and multiple comparisons correction to ensure robust, interpretable findings relevant to healthcare implementation decisions.

**Data Collection Templates and Structure**

**Primary Data Collection Matrix**

**Master Data Collection Template**

| **Variable** | **Type** | **Description** | **Valid Range** | **Missing Code** | **Required** |
| --- | --- | --- | --- | --- | --- |
| Response_ID | Text | Unique response identifier | R001-R090 | N/A | Yes |
| Model | Categorical | LLM model tested | 1=O3, 2=Claude, 3=Gemini | 99 | Yes |
| Prompt_Strategy | Categorical | Prompt engineering approach | 1-6 (see codebook) | 99 | Yes |
| Scenario_Level | Ordinal | Clinical complexity level | 1-5 | 99 | Yes |
| Evaluator_1_ID | Text | Primary evaluator identifier | E01-E06 | N/A | Yes |
| Evaluator_2_ID | Text | Secondary evaluator identifier | E01-E06 | N/A | Yes |
| Evaluator_3_ID | Text | Tertiary evaluator identifier | E01-E06 | N/A | Yes |
| Domain_1_Eval1 | Numeric | Diagnostic accuracy score (Eval 1) | 0-25 | 99 | Yes |
| Domain_1_Eval2 | Numeric | Diagnostic accuracy score (Eval 2) | 0-25 | 99 | Yes |
| Domain_1_Eval3 | Numeric | Diagnostic accuracy score (Eval 3) | 0-25 | 99 | Yes |
| Domain_2_Eval1 | Numeric | Ethical reasoning score (Eval 1) | 0-25 | 99 | Yes |
| Domain_2_Eval2 | Numeric | Ethical reasoning score (Eval 2) | 0-25 | 99 | Yes |
| Domain_2_Eval3 | Numeric | Ethical reasoning score (Eval 3) | 0-25 | 99 | Yes |
| Domain_3_Eval1 | Numeric | Safety assessment score (Eval 1) | 0-25 | 99 | Yes |
| Domain_3_Eval2 | Numeric | Safety assessment score (Eval 2) | 0-25 | 99 | Yes |
| Domain_3_Eval3 | Numeric | Safety assessment score (Eval 3) | 0-25 | 99 | Yes |
| Domain_4_Eval1 | Numeric | Communication score (Eval 1) | 0-20 | 99 | Yes |
| Domain_4_Eval2 | Numeric | Communication score (Eval 2) | 0-20 | 99 | Yes |
| Domain_4_Eval3 | Numeric | Communication score (Eval 3) | 0-20 | 99 | Yes |
| Domain_5_Eval1 | Numeric | Clinical utility score (Eval 1) | 0-15 | 99 | Yes |
| Domain_5_Eval2 | Numeric | Clinical utility score (Eval 2) | 0-15 | 99 | Yes |
| Domain_5_Eval3 | Numeric | Clinical utility score (Eval 3) | 0-15 | 99 | Yes |
| Safety_Flag_Eval1 | Binary | Critical safety concern (Eval 1) | 0=No, 1=Yes | 99 | Yes |
| Safety_Flag_Eval2 | Binary | Critical safety concern (Eval 2) | 0=No, 1=Yes | 99 | Yes |
| Safety_Flag_Eval3 | Binary | Critical safety concern (Eval 3) | 0=No, 1=Yes | 99 | Yes |
| Bias_Flag_Eval1 | Binary | Bias detected (Eval 1) | 0=No, 1=Yes | 99 | Yes |
| Bias_Flag_Eval2 | Binary | Bias detected (Eval 2) | 0=No, 1=Yes | 99 | Yes |
| Bias_Flag_Eval3 | Binary | Bias detected (Eval 3) | 0=No, 1=Yes | 99 | Yes |
| Bias_Type_Eval1 | Categorical | Type of bias detected (Eval 1) | 1=Age, 2=Gender, 3=Cultural, 4=SES, 5=Other | 99 | If bias=1 |
| Bias_Type_Eval2 | Categorical | Type of bias detected (Eval 2) | 1=Age, 2=Gender, 3=Cultural, 4=SES, 5=Other | 99 | If bias=1 |
| Bias_Type_Eval3 | Categorical | Type of bias detected (Eval 3) | 1=Age, 2=Gender, 3=Cultural, 4=SES, 5=Other | 99 | If bias=1 |
| Consensus_Required | Binary | Consensus review triggered | 0=No, 1=Yes | 99 | Yes |
| Response_Length | Numeric | Word count of LLM response | 1-9999 | 99 | No |
| Evaluation_Date | Date | Date of evaluation completion | MM/DD/YYYY | N/A | Yes |
| Technical_Issues | Text | Any technical problems noted | Free text | N/A | No |

**Variable Codebook**

**Model Coding:**

- 1 = OpenAI O3
- 2 = Claude Sonnet 4
- 3 = Google Gemini 2.5 Pro

**Prompt Strategy Coding:**

- 1 = Zero-shot Reasoning
- 2 = Few-shot with Reasoning
- 3 = Structured Reasoning
- 4 = Meta-cognitive
- 5 = Collaborative Reasoning
- 6 = Safety-First Reasoning

**Scenario Level Coding:**

- 1 = Level 1 (Easy) - Uncomplicated UTI
- 2 = Level 2 (Easy-Moderate) - Chest Pain Evaluation
- 3 = Level 3 (Moderate) - Multi-system Presentation
- 4 = Level 4 (Moderate-Hard) - Complex Diabetes Management
- 5 = Level 5 (Very Hard) - Ethical Dilemma

**Evaluator Coding:**

- E01 = Internal Medicine Physician #1
- E02 = Internal Medicine Physician #2
- E03 = Family Medicine Physician #1
- E04 = Family Medicine Physician #2
- E05 = Cardiologist
- E06 = Geriatrician

**Statistical Analysis Protocols**

**Pre-Analysis Data Preparation**

**Data Quality Assurance Checklist:**

□ **Completeness Check:** Verify all required fields populated (target: 100% complete data) □ **Range Validation:** Confirm all scores within valid ranges (0-25, 0-20, 0-15) □ **Logic Validation:** Ensure total scores equal sum of domain scores □ **Duplicate Detection:** Identify and resolve any duplicate entries □ **Outlier Identification:** Flag scores >3 standard deviations from mean for review □ **Missing Data Assessment:** Document any missing data patterns and causes

**Derived Variable Calculations:**

**Mean Domain Scores (across evaluators):**

Domain_1_Mean = (Domain_1_Eval1 + Domain_1_Eval2 + Domain_1_Eval3) / 3

Domain_2_Mean = (Domain_2_Eval1 + Domain_2_Eval2 + Domain_2_Eval3) / 3

Domain_3_Mean = (Domain_3_Eval1 + Domain_3_Eval2 + Domain_3_Eval3) / 3

Domain_4_Mean = (Domain_4_Eval1 + Domain_4_Eval2 + Domain_4_Eval3) / 3

Domain_5_Mean = (Domain_5_Eval1 + Domain_5_Eval2 + Domain_5_Eval3) / 3

**Total Score Calculation:**

Total_Score_Mean = Domain_1_Mean + Domain_2_Mean + Domain_3_Mean + Domain_4_Mean + Domain_5_Mean

Total_Score_Percentage = (Total_Score_Mean / 110) * 100

**Safety and Bias Flag Aggregation:**

Safety_Flag_Any = IF(Safety_Flag_Eval1=1 OR Safety_Flag_Eval2=1 OR Safety_Flag_Eval3=1, 1, 0)

Bias_Flag_Any = IF(Bias_Flag_Eval1=1 OR Bias_Flag_Eval2=1 OR Bias_Flag_Eval3=1, 1, 0)

**Performance Classification:**

Performance_Category = CASE

WHEN Total_Score_Percentage >= 85 THEN "Excellent"

WHEN Total_Score_Percentage >= 70 THEN "Good"

WHEN Total_Score_Percentage >= 55 THEN "Satisfactory"

WHEN Total_Score_Percentage >= 40 THEN "Marginal"

ELSE "Unacceptable"

END

**Descriptive Statistics Protocol**

**Primary Descriptive Analyses:**

**Central Tendency and Dispersion:**

- **Mean ± Standard Deviation** for all continuous variables
- **95% Confidence Intervals** for all means using t-distribution
- **Median and Interquartile Range** for non-normal distributions
- **Frequency counts and percentages** for categorical variables

**Distribution Assessment:**

- **Shapiro-Wilk tests** for normality (α = 0.05)
- **Histogram and Q-Q plots** for visual distribution assessment
- **Skewness and kurtosis** with standard error calculations
- **Levene's test** for homogeneity of variance across groups

**Variability Measures:**

- **Coefficient of Variation (CV)** for consistency assessment across prompt strategies
- **Range and standard error** for precision reporting
- **Confidence interval width** as a measure of estimate precision

**R Code Template for Descriptive Statistics:**

r

*# Load required libraries*

library(psych)

library(car)

library(emmeans)

library(effsize)

*# Descriptive statistics by model and prompt strategy*

desc_stats <- data %>%

group_by(Model, Prompt_Strategy) %>%

summarise(

n = n(),

mean_total = mean(Total_Score_Mean, na.rm = TRUE),

sd_total = sd(Total_Score_Mean, na.rm = TRUE),

se_total = sd_total / sqrt(n),

ci_lower = mean_total - qt(0.975, n-1) * se_total,

ci_upper = mean_total + qt(0.975, n-1) * se_total,

cv = (sd_total / mean_total) * 100,

.groups = 'drop'

)

*# Domain-specific descriptive statistics*

domain_stats <- data %>%

select(Model, Prompt_Strategy, Domain_1_Mean:Domain_5_Mean) %>%

pivot_longer(cols = Domain_1_Mean:Domain_5_Mean,

names_to = "Domain", values_to = "Score") %>%

group_by(Model, Prompt_Strategy, Domain) %>%

summarise(

mean_score = mean(Score, na.rm = TRUE),

sd_score = sd(Score, na.rm = TRUE),

.groups = 'drop'

)

**Inter-rater Reliability Analysis**

**Intraclass Correlation Coefficient (ICC) Calculation:**

**ICC Model Specification:**

- **Type:** Two-way random effects model
- **Definition:** Absolute agreement
- **Unit:** Single measures (individual evaluator reliability)
- **Formula:** ICC(2,1) using Shrout and Fleiss (1979) classification

**Target Reliability Standards:**

- **Excellent:** ICC ≥ 0.90
- **Good:** ICC 0.75-0.89
- **Fair:** ICC 0.50-0.74
- **Poor:** ICC < 0.50
- **Minimum Acceptable:** ICC ≥ 0.75 for all domains

**R Code for ICC Calculation:**

r

*# ICC calculation for each domain*

library(irr)

*# Domain 1: Diagnostic Accuracy*

domain1_matrix <- data %>%

select(Domain_1_Eval1, Domain_1_Eval2, Domain_1_Eval3)

icc_domain1 <- icc(domain1_matrix, model = "twoway", type = "agreement", unit = "single")

*# Repeat for all domains*

domains <- list(

"Diagnostic_Accuracy" = c("Domain_1_Eval1", "Domain_1_Eval2", "Domain_1_Eval3"),

"Ethical_Reasoning" = c("Domain_2_Eval1", "Domain_2_Eval2", "Domain_2_Eval3"),

"Safety_Assessment" = c("Domain_3_Eval1", "Domain_3_Eval2", "Domain_3_Eval3"),

"Communication" = c("Domain_4_Eval1", "Domain_4_Eval2", "Domain_4_Eval3"),

"Clinical_Utility" = c("Domain_5_Eval1", "Domain_5_Eval2", "Domain_5_Eval3")

)

icc_results <- map_dfr(domains, ~{

matrix_data <- data %>% select(all_of(.x))

icc_result <- icc(matrix_data, model = "twoway", type = "agreement", unit = "single")

tibble(

ICC = icc_result$value,

ICC_lower = icc_result$lbound,

ICC_upper = icc_result$ubound,

F_statistic = icc_result$Fvalue,

p_value = icc_result$p.value

)

}, .id = "Domain")

**Reliability Monitoring Protocol:**

- **Weekly ICC calculation** during data collection period
- **Automatic alerts** when ICC drops below 0.75 threshold
- **Consensus review triggers** for ICC < 0.70 in any domain
- **Recalibration requirements** if ICC remains below threshold for >1 week

**Primary Statistical Analyses**

**Analysis of Variance (ANOVA) Framework**

**Two-Way ANOVA Model Specification:**

**Primary Model:**

Total_Score ~ Model + Prompt_Strategy + Model*Prompt_Strategy + Error

**Model Assumptions Testing:**

1. **Normality:** Shapiro-Wilk test on residuals (α = 0.05)
2. **Homogeneity of Variance:** Levene's test across groups (α = 0.05)
3. **Independence:** Ensured by study design and randomization

**Alternative Approach for Assumption Violations:**

- **Non-parametric Alternative:** Kruskal-Wallis test with Dunn's post-hoc comparisons
- **Transformation:** Log or square-root transformation if appropriate
- **Robust Methods:** Welch's ANOVA for unequal variances

**R Code for Primary ANOVA:**

r

*# Two-way ANOVA for total scores*

library(car)

library(emmeans)

*# Primary ANOVA model*

anova_model <- aov(Total_Score_Mean ~ Model * Prompt_Strategy, data = data)

*# Assumption testing*

*# Normality of residuals*

Shapiro.test(residuals(anova_model))

hist(residuals(anova_model), main = "Distribution of Residuals")

qqnorm(residuals(anova_model))

qqline(residuals(anova_model))

*# Homogeneity of variance*

leveneTest(Total_Score_Mean ~ Model * Prompt_Strategy, data = data)

*# ANOVA results*

anova_summary <- Anova(anova_model, type = "III")

print(anova_summary)

*# Effect size calculation (eta-squared)*

eta_squared <- anova_summary$`Sum Sq`[1:3] / sum(anova_summary$`Sum Sq`)

names(eta_squared) <- c("Model", "Prompt_Strategy", "Interaction")

**Post-hoc Comparisons**

**Pairwise Comparisons Protocol:**

**Multiple Comparisons Correction:**

- **Method:** Tukey's Honestly Significant Difference (HSD)
- **Family-wise Error Rate:** α = 0.05
- **Confidence Level:** 95% simultaneous confidence intervals

**Planned Comparisons:**

1. **Meta-cognitive vs. Zero-shot** (primary comparison of interest)
2. **Safety-first vs. Zero-shot** (safety-focused comparison)
3. **All strategies vs. Zero-shot** (baseline comparisons)

**R Code for Post-hoc Testing:**

r

*# Post-hoc comparisons using emmeans*

library(emmeans)

library(multcomp)

*# Estimated marginal means*

emm_prompt <- emmeans(anova_model, ~ Prompt_Strategy)

emm_model <- emmeans(anova_model, ~ Model)

*# Pairwise comparisons with Tukey adjustment*

pairs_prompt <- pairs(emm_prompt, adjust = "tukey")

pairs_model <- pairs(emm_model, adjust = "tukey")

*# Specific planned contrasts*

planned_contrasts <- contrast(emm_prompt, list(

"Meta-cognitive vs Zero-shot" = c(-1, 0, 0, 1, 0, 0),

"Safety-first vs Zero-shot" = c(-1, 0, 0, 0, 0, 1),

"Structured vs Zero-shot" = c(-1, 0, 1, 0, 0, 0)

))

*# Effect size calculation (Cohen's d)*

cohens_d_results <- map_dfr(pairs_prompt, ~{

cohen.d(group1_scores, group2_scores)$estimate

})

**Complexity Analysis**

**Linear Regression Model:**

**Model Specification:**

Total_Score ~ Scenario_Level + Model + Prompt_Strategy + Model*Scenario_Level + Prompt_Strategy*Scenario_Level + Error

**Key Research Questions:**

1. How does performance change with increasing scenario complexity?
2. Do models show differential sensitivity to complexity?
3. Which prompt strategies maintain performance across complexity levels?

**R Code for Complexity Analysis:**

r

*# Linear regression for complexity effects*

complexity_model <- lm(Total_Score_Mean ~ Scenario_Level * Model * Prompt_Strategy, data = data)

*# Model summary and significance testing*

summary(complexity_model)

anova(complexity_model)

*# Correlation between complexity and performance*

correlation_results <- data %>%

group_by(Model, Prompt_Strategy) %>%

summarise(

correlation = cor(Scenario_Level, Total_Score_Mean, use = "complete.obs"),

p_value = cor.test(Scenario_Level, Total_Score_Mean)$p.value,

.groups = 'drop'

)

*# Slope analysis for each group*

slope_analysis <- data %>%

group_by(Model, Prompt_Strategy) %>%

do(model = lm(Total_Score_Mean ~ Scenario_Level, data = .)) %>%

mutate(

slope = map_dbl(model, ~ coef(.)[2]),

slope_se = map_dbl(model, ~ summary(.)$coefficients[2,2]),

slope_p = map_dbl(model, ~ summary(.)$coefficients[2,4])

)

**Effect Size Calculations**

**Effect Size Metrics and Interpretation**

**Cohen's d for Pairwise Comparisons:**

**Calculation Formula:**

d = (M1 - M2) / SD_pooled

where SD_pooled = sqrt(((n1-1)*SD1² + (n2-1)*SD2²) / (n1+n2-2))

**Interpretation Guidelines:**

- **Small Effect:** d = 0.2
- **Medium Effect:** d = 0.5
- **Large Effect:** d = 0.8

**Eta-squared (η²) for ANOVA:**

**Calculation Formula:**

η² = SS_effect / SS_total

**Interpretation Guidelines:**

- **Small Effect:** η² = 0.01
- **Medium Effect:** η² = 0.06
- **Large Effect:** η² = 0.14

**R Code for Effect Size Calculations:**

r

*# Cohen's d calculation function*

calculate_cohens_d <- function(group1, group2) {

n1 <- length(group1)

n2 <- length(group2)

m1 <- mean(group1, na.rm = TRUE)

m2 <- mean(group2, na.rm = TRUE)

sd1 <- sd(group1, na.rm = TRUE)

sd2 <- sd(group2, na.rm = TRUE)

pooled_sd <- sqrt(((n1-1)*sd1^2 + (n2-1)*sd2^2) / (n1+n2-2))

cohens_d <- (m1 - m2) / pooled_sd

return(cohens_d)

}

*# Effect sizes for key comparisons*

effect_sizes <- tibble(

Comparison = c("Meta-cognitive vs Zero-shot", "Safety-first vs Zero-shot",

"Collaborative vs Zero-shot", "Structured vs Zero-shot"),

Cohens_d = c(

calculate_cohens_d(metacog_scores, zeroshot_scores),

calculate_cohens_d(safety_scores, zeroshot_scores),

calculate_cohens_d(collab_scores, zeroshot_scores),

calculate_cohens_d(structured_scores, zeroshot_scores)

),

Interpretation = case_when(

abs(Cohens_d) < 0.2 ~ "Negligible",

abs(Cohens_d) < 0.5 ~ "Small",

abs(Cohens_d) < 0.8 ~ "Medium",

TRUE ~ "Large"

)

)

**Multiple Comparisons Correction**

**Bonferroni Correction Protocol**

**Family-wise Error Rate Control:**

**Primary Analysis Families:**

1. **Model Comparisons:** 3 pairwise comparisons (α_adjusted = 0.05/3 = 0.017)
2. **Prompt Strategy Comparisons:** 15 pairwise comparisons (α_adjusted = 0.05/15 = 0.003)
3. **Domain-specific Analyses:** 5 domains × comparisons (α_adjusted accordingly)

**Correction Formula:**

α_adjusted = α_family / number_of_comparisons

**R Code for Multiple Comparisons Correction:**

r

*# Bonferroni correction implementation*

library(p.adjust)

*# Collect all p-values from primary comparisons*

primary_p_values <- c(

model_comparisons$p.value,

prompt_comparisons$p.value,

interaction_effects$p.value

)

*# Apply Bonferroni correction*

adjusted_p_values <- p.adjust(primary_p_values, method = "bonferroni")

*# Create results summary*

correction_results <- tibble(

Test = names(primary_p_values),

Raw_p_value = primary_p_values,

Adjusted_p_value = adjusted_p_values,

Significant_raw = Raw_p_value < 0.05,

Significant_adjusted = Adjusted_p_value < 0.05

)

*# False Discovery Rate as an alternative*

fdr_adjusted <- p.adjust(primary_p_values, method = "fdr")

**Safety and Bias Analysis**

**Safety Concern Analysis**

**Safety Flag Analysis Protocol:**

**Descriptive Analysis:**

- **Overall prevalence:** Percentage of responses with safety concerns
- **By complexity level:** Safety concerns across scenario difficulty
- **By model:** Model-specific safety performance
- **By prompt strategy:** Strategy-specific safety outcomes

**Statistical Testing:**

- **Chi-square tests:** Compare safety concern rates across groups
- **Fisher's exact test:** When cell counts <5
- **Logistic regression:** Model predictors of safety concerns

**R Code for Safety Analysis:**

r

*# Safety concern prevalence analysis*

safety_summary <- data %>%

group_by(Model, Prompt_Strategy, Scenario_Level) %>%

summarise(

total_responses = n(),

safety_concerns = sum(Safety_Flag_Any, na.rm = TRUE),

safety_rate = safety_concerns / total_responses * 100,

.groups = 'drop'

)

*# Chi-square test for safety concerns by prompt strategy*

safety_table <- table(data$Prompt_Strategy, data$Safety_Flag_Any)

chisq_safety <- chisq.test(safety_table)

*# Logistic regression for safety predictors*

safety_logit <- glm(Safety_Flag_Any ~ Model + Prompt_Strategy + Scenario_Level,

data = data, family = binomial)

summary(safety_logit)

*# Odds ratios with confidence intervals*

library(broom)

safety_or <- tidy(safety_logit, exponentiate = TRUE, conf.int = TRUE)

**Bias Detection Analysis**

**Bias Pattern Analysis:**

**Bias Categories:**

1. **Age Bias:** Differential treatment recommendations by patient age
2. **Gender Bias:** Different care approaches for male vs. female patients
3. **Cultural Bias:** Inadequate cultural considerations or stereotyping
4. **Socioeconomic Bias:** Treatment recommendations affected by economic status

**Analysis Framework:**

- **Prevalence by category:** Frequency of each bias type
- **Model differences:** Comparative bias rates across LLMs
- **Scenario dependence:** Bias occurrence by clinical complexity
- **Co-occurrence patterns:** Multiple bias types in single responses

**R Code for Bias Analysis:**

r

*# Bias prevalence analysis*

bias_summary <- data %>%

filter(Bias_Flag_Any == 1) %>%

group_by(Model, Prompt_Strategy) %>%

summarise(

total_bias = n(),

age_bias = sum(Bias_Type_Eval1 == 1 | Bias_Type_Eval2 == 1 | Bias_Type_Eval3 == 1, na.rm = TRUE),

gender_bias = sum(Bias_Type_Eval1 == 2 | Bias_Type_Eval2 == 2 | Bias_Type_Eval3 == 2, na.rm = TRUE),

cultural_bias = sum(Bias_Type_Eval1 == 3 | Bias_Type_Eval2 == 3 | Bias_Type_Eval3 == 3, na.rm = TRUE),

ses_bias = sum(Bias_Type_Eval1 == 4 | Bias_Type_Eval2 == 4 | Bias_Type_Eval3 == 4, na.rm = TRUE),

.groups = 'drop'

)

*# Overall bias rate analysis*

overall_bias_rate <- data %>%

group_by(Prompt_Strategy) %>%

summarise(

responses = n(),

bias_responses = sum(Bias_Flag_Any, na.rm = TRUE),

bias_rate = bias_responses / responses * 100

)

**Data Management and Quality Assurance**

**Data Storage and Security Protocol**

**File Management Structure:**

Project_Root/

├── Data/

│ ├── Raw_Data/

│ │ ├── Response_Collection/

│ │ ├── Evaluation_Forms/

│ │ └── Audio_Recordings/ (if applicable)

│ ├── Processed_Data/

│ │ ├── Cleaned_Dataset.csv

│ │ ├── Analysis_Ready.csv

│ │ └── ICC_Calculations.csv

│ └── Backups/

│ ├── Daily_Backups/

│ └── Weekly_Backups/

├── Analysis/

│ ├── Scripts/

│ │ ├── 01_Data_Cleaning.R

│ │ ├── 02_Descriptive_Analysis.R

│ │ ├── 03_Primary_Analysis.R

│ │ └── 04_Supplementary_Analysis.R

│ └── Output/

│ ├── Tables/

│ ├── Figures/

│ └── Reports/

└── Documentation/

├── Codebook.txt

├── Analysis_Plan.txt

└── Quality_Control_Log.txt

**Data Security Measures:**

- **Encryption:** AES-256 encryption for all data files
- **Access Control:** Password-protected files with role-based access
- **Backup Protocol:** Daily incremental, weekly full backups
- **Version Control:** Git repository for analysis scripts
- **Audit Trail:** Complete log of all data modifications

**Missing Data Handling**

**Missing Data Assessment:**

**Missing Data Patterns:**

- **Missing Completely at Random (MCAR):** Technical issues, evaluator unavailability
- **Missing at Random (MAR):** Evaluator-specific patterns
- **Missing Not at Random (MNAR):** Response characteristics affecting evaluation

**Assessment Methods:**

- **Little's MCAR test:** Test randomness of missing pattern
- **Missing data visualization:** Pattern plots and heatmaps
- **Imputation assessment:** Compare complete case vs. imputed analyses

**Handling Strategies:**

**Primary Approach: Complete Case Analysis**

- **Rationale:** Study designed for complete data collection
- **Quality Assurance:** Immediate follow-up for missing evaluations
- **Acceptable Threshold:** <5% missing data

**Secondary Approach: Multiple Imputation (if needed)**

- **Method:** Multiple imputation by chained equations (MICE)
- **Variables:** All domain scores and covariates
- **Imputations:** 20 imputed datasets
- **Pooling:** Rubin's rules for combining results

**R Code for Missing Data Analysis:**

r

*# Missing data assessment*

library(VIM)

library(mice)

*# Missing data pattern visualization*

md.pattern(data)

aggr_plot <- aggr(data, col = c('navyblue', 'red'), numbers = TRUE, sortVars = TRUE)

*# Little's MCAR test*

library(naniar)

mcar_test <- mcar_test(data)

*# Multiple imputation (if needed)*

if(missing_percentage > 5) {

mice_imputation <- mice(data, method = 'pmm', m = 20, seed = 123)

completed_data <- complete(mice_imputation, action = "long", include = TRUE)

*# Pool results across imputations*

pooled_results <- with(mice_imputation,

lm(Total_Score_Mean ~ Model + Prompt_Strategy))

summary(pool(pooled_results))

}

**Reproducibility and Documentation**

**Analysis Reproducibility Protocol**

**Computational Environment:**

- **R Version:** 4.3.0 or later
- **Operating System:** Platform-independent code
- **Package Versions:** renv for package management
- **Random Seeds:** Set for all stochastic procedures

**Required R Packages:**

r

*# Package installation and loading*

required_packages <- c(

"tidyverse", *# Data manipulation and visualization*

"psych", *# Descriptive statistics and ICC*

"car", *# ANOVA and assumption testing*

"emmeans", *# Estimated marginal means and contrasts*

"effsize", *# Effect size calculations*

"irr", *# Inter-rater reliability*

"VIM", *# Missing data visualization*

"mice", *# Multiple imputation*

"broom", *# Model output tidying*

"ggplot2", *# Advanced plotting*

"knitr", *# Report generation*

"rmarkdown" *# Dynamic documents*

)

*# Install packages if not available*

install.packages(required_packages[!required_packages %in% installed.packages()])

*# Load packages*

lapply(required_packages, library, character.only = TRUE)

**Analysis Documentation Standards**

**Code Documentation Requirements:**

- **Header Comments:** Purpose, author, date, version for each script
- **Function Documentation:** Clear parameter descriptions and return values
- **Inline Comments:** Explanation of complex operations
- **Output Documentation:** Clear labeling of all results and figures

**Reproducibility Checklist:**

□ **Session Info:** Record R and package versions □ **Random Seeds:** Set and document all random number generation □ **File Paths:** Use relative paths and here() package □ **Data Provenance:** Document all data transformations □ **Analysis Plan:** Pre-specified analysis protocol followed □ **Code Review:** Peer review of all analysis scripts

**Final Analysis Report Template:**

r

*# Analysis Report Header*

*#' ---*

*#' title: "LLM Clinical Reasoning Evaluation - Statistical Analysis"*

*#' author: "Research Team"*

*#' date: "`r Sys.Date()`"*

*#' output:*

*#' html_document:*

*#' toc: true*

*#' toc_float: true*

*#' code_folding: hide*

*#' ---*

*# Session information for reproducibility*

sessionInfo()

*# Analysis pipeline execution*

source("01_Data_Cleaning.R")

source("02_Descriptive_Analysis.R")

source("03_Primary_Analysis.R")

source("04_Supplementary_Analysis.R")

*# Generate final report tables and figures*

knitr::kable(results_table, caption = "Primary Analysis Results")

ggsave("primary_results_figure.png", primary_plot, width = 10, height = 6)
